# Circulating Tumor DNA as a Minimal Residual Disease Assessment and Recurrence Risk in Patients Undergoing Curative-Intent Resection with or without Adjuvant Chemotherapy in Colorectal Cancer: A Systematic Review and Meta-Analysis

**DOI:** 10.3390/ijms241210230

**Published:** 2023-06-16

**Authors:** Anusha Chidharla, Eliot Rapoport, Kriti Agarwal, Samragnyi Madala, Brenda Linares, Weijing Sun, Sakti Chakrabarti, Anup Kasi

**Affiliations:** 1 Department of Medical Oncology, University of Kansas Cancer Center, Kansas City, KS 66205, USA; wsun2@kumc.edu; 2 Department of Internal Medicine, Montefiore Albert Einstein College of Medicine, Bronx, NY 10461, USA; erapoport@montefiore.org; 3 Department of Internal Medicine, Hackensack University Medical Center, Hackensack, NJ 07601, USA; kritiagarwalmd@gmail.com; 4 Department of Medical Oncology, University of Iowa Hospitals and Clinics, Iowa City, IA 55224, USA; sam.madala@gmail.com; 5 Research and Learning Department, Kansas University Medical Center, Kansas City, KS 66211, USA; blinares@kumc.edu; 6 Department of Medical Oncology, University Hospitals Seidman Cancer Center, Cleveland, OH 44106, USA; sakti.chakrabarti@uhhospitals.org

**Keywords:** circulating tumor DNA, minimal residual disease, curative-intent surgery, adjuvant chemotherapy, colorectal cancer, recurrence-free survival

## Abstract

Emerging data have suggested that circulating tumor DNA (ctDNA) can be a reliable biomarker for minimal residual disease (MRD) in CRC patients. Recent studies have shown that the ability to detect MRD using ctDNA assay after curative-intent surgery will change how to assess the recurrence risk and patient selection for adjuvant chemotherapy. We performed a meta-analysis of post-operative ctDNA in stage I–IV (oligometastatic) CRC patients after curative-intent resection. We included 23 studies representing 3568 patients with evaluable ctDNA in CRC patient post-curative-intent surgery. Data were extracted from each study to perform a meta-analysis using RevMan 5.4. software. Subsequent subgroup analysis was performed for stages I–III and oligometastatic stage IV CRC patients. Results showed that the pooled hazard ratio (HR) for recurrence-free survival (RFS) in post-surgical ctDNA-positive versus -negative patients in all stages was 7.27 (95% CI 5.49–9.62), *p* < 0.00001. Subgroup analysis revealed pooled HRs of 8.14 (95% CI 5.60–11.82) and 4.83 (95% CI 3.64–6.39) for stages I–III and IV CRC, respectively. The pooled HR for RFS in post-adjuvant chemotherapy ctDNA-positive versus -negative patients in all stages was 10.59 (95% CI 5.59–20.06), *p* < 0.00001. Circulating tumor DNA (ctDNA) analysis has revolutionized non-invasive cancer diagnostics and monitoring, with two primary forms of analysis emerging: tumor-informed techniques and tumor-agnostic or tumor-naive techniques. Tumor-informed methods involve the initial identification of somatic mutations in tumor tissue, followed by the targeted sequencing of plasma DNA using a personalized assay. In contrast, the tumor-agnostic approach performs ctDNA analysis without prior knowledge of the patient’s tumor tissue molecular profile. This review highlights the distinctive features and implications of each approach. Tumor-informed techniques enable the precise monitoring of known tumor-specific mutations, leveraging the sensitivity and specificity of ctDNA detection. Conversely, the tumor-agnostic approach allows for a broader genetic and epigenetic analysis, potentially revealing novel alterations and enhancing our understanding of tumor heterogeneity. Both approaches have significant implications for personalized medicine and improved patient outcomes in the field of oncology. The subgroup analysis based on the ctDNA method showed pooled HRs of 8.66 (95% CI 6.38–11.75) and 3.76 (95% CI 2.58–5.48) for tumor-informed and tumor-agnostic, respectively. Our analysis emphasizes that post-operative ctDNA is a strong prognostic marker of RFS. Based on our results, ctDNA can be a significant and independent predictor of RFS. This real-time assessment of treatment benefits using ctDNA can be used as a surrogate endpoint for the development of novel drugs in the adjuvant setting.

## 1. Introduction

Cell-free DNA (cfDNA) are small DNA fragments (160–200 bp) released into the bloodstream during cell death. In healthy adults, cfDNA is primarily released by hematopoietic cells; however, in the setting of cancer, many tumors also release DNA fragments, referred to as circulating tumor DNA (ctDNA), into the systemic circulation [1,2,3]. CtDNA has a short half-life of approximately 2 h. This property allows it to be used as a dynamic marker for tracking the presence of the tumor [4,5]. Although somewhat limited by the delayed turnaround time and cost, there is significant interest in ctDNA. It is a minimally invasive test, which, given its dynamic nature, has high sensitivity and specificity [1,6,7].

Two forms of ctDNA analysis have been developed: tumor-informed techniques and tumor-agnostic or tumor-naive techniques. In tumor-informed methods (e.g., Signatera and Safe-SeqS), somatic mutations are first identified in tumor tissue, followed by the targeted sequencing of plasma DNA using a personalized assay. In the tumor-agnostic approach, ctDNA analysis is performed without the knowledge of the patient’s tumor tissue molecular profile (e.g., Guardant Reveal assay) [3,8,9]. A significant drawback is the prolonged turnaround time required for personalization. Nevertheless, both methods are currently being evaluated despite cost concerns, hematopoiesis-associated false positives, and reproducibility.

The utility of ctDNA is being explored in numerous contexts, with evidence supporting its role in early cancer detection, monitoring treatment response, and evaluating recurrence and efficacy for multiple forms of cancer. Minimal residual disease (MRD) is defined as micro-metastases that are still present after definitive treatment, such as surgery or post-adjuvant systemic therapy. The prognostic role of ctDNA-based MRD detection is established in various hematologic malignancies and incorporated into standard management guidelines [10,11]. One specific area of interest is its role in assessing MRD and the possibility of its use to guide therapeutic decisions. One hope is that it will be able to guide treatment in the controversial setting of adjuvant chemotherapy (ACT) in stage II and other non- or oligometastatic colorectal cancers (CRCs). The role of ACT in this setting is poorly defined because of the heterogeneity within disease stages [12].

Colorectal cancer (CRC) is the third most common cancer in the United States, affecting both males and females. CRC is the second leading cause of cancer-related deaths in the U.S. and worldwide. The lifetime risk of developing colorectal cancer is around 1 in 25 (4.0%) for females and 1 in 23 (4.3%) for males. One challenge with the treatment of colon cancer is its high recurrence rate. The risk of recurrence remains high at 20–30% in localized and locally advanced cancers. Because of this, better tools are needed for the early detection of recurrence and presence of disease. 

The benefit of adjuvant 5-FU-based chemotherapy in locally advanced colon cancer has been recognized since the late 1980s. A meta-analysis published by Buyse et al. in 1988 comparing adjuvant 5-FU with surgery alone favored adjuvant chemotherapy, with a mortality odds ratio of 0.83 (95% CI 0.70–0.98) [13]. This was established by North Central Cancer Therapy Group (NCCTG)2 and Intergroup (INT)-00353 trials, which formed the basis for current guideline recommendations to include 5-FU-based adjuvant chemotherapy in stage II/III colon cancer patients. While the guidelines for adjuvant chemotherapy in stage III colon cancer are unambiguous, its use in stage II disease is debatable—especially considering the toxicity associated with chemotherapy regimens with unclear benefits [13,14]. Current guidelines recommend 3–6 months of adjuvant chemotherapy after surgery for nonmetastatic colon cancer [15]. The role of adjuvant chemotherapy (ACT) in stage II colon cancer is controversial given the heterogeneity within disease stages—not all stage II colon cancer patients need adjuvant chemotherapy. In patients deemed to have high-risk stage II CRC, surgery is followed by adjuvant chemotherapy. This decision is made based on tumor size as well as the pathological and clinical features of the disease, which are relatively poor predictors [15]. Not all patients require ACT, and it has been challenging to determine what subset does [12,13,16,17]. Henceforth, there is a need for predictive and prognostic biomarkers for the follow-up detection of early recurrence, thereby enabling appropriate follow-up and therapeutic strategies for early recurrence detection and curative treatment. 

Recent advances in technology in ctDNA assay can detect minimal residual disease (MRD) after curative-intent surgery [18,19]. Using ctDNA to guide the treatment can help avoid the toxic effects of chemotherapy after surgery, especially in patients with a low risk of recurrence. ctDNA has been shown to have a prognostic value and is a good predictor of cancer recurrence in many recent studies [20]. Emerging data have suggested that circulating tumor DNA (ctDNA) can be a reliable biomarker for MRD. This may change how to assess the recurrence risk and patient selection for adjuvant chemotherapy [21]. Therefore, we conducted a systematic review and meta-analysis of studies evaluating the value of ctDNA in the post-surgical and post-adjuvant chemotherapy periods to predict prognosis and recurrence.

## 2. Methods

This systematic review and meta-analysis was exempt from institutional review board approval based on Kansas University Medical Center criteria. The study was conducted in accordance with the Preferred Reporting Items for Systematic Reviews and Meta-analyses (PRISMA, PRISMA_2020_checklist.pdf (prisma-statement.org) https://www.equator-network.org/reporting-guidelines/prisma/) (accessed on 2 April 2023) recommendations.

A professional librarian searched PubMed/Medline, EMBASE, Web of Science, Cochrane Library, and Google from the database inception through to 8 June 2022, using Keywords, Medical Subject Heading (MeSH), and EMTREE subject headings to search for the concepts of colon cancer, ctDNA, survival, and types of studies. The search included full-text articles and conference presentations. The search terms colon cancer, rectal cancer, ctDNA, colorectal cancer, circulating tumor DNA, recurrence-free survival, post-surgery, and post-adjuvant chemotherapy were expanded and used with appropriate MeSH terms. The results were refined according to the study type and outcomes. 

### 2.1. Study Eligibility

Studies were evaluated by at least two independent reviewers (AC, ER, KA), with a third confirming the final inclusion and resolving disagreements (AK). Studies were chosen on the basis of the following criteria: (1) randomized clinical trials or prospective/retrospective cohort studies; (2) patients with stage I–III or oligometastatic stage IV colorectal cancer; (3) studies examining post-operative ctDNA status or post-adjuvant ctDNA status; (4) ctDNA data were derived from a panel of mutations rather than single mutations; (5) data were available on patient outcomes, including disease-free survival, recurrence-free survival, or overall survival; (6) data were not better represented in another entry; (7) both full published manuscripts and conference abstracts were included. Studies beyond the inclusion criteria or those originally published in a language other than English were excluded. 

### 2.2. Data Extraction

Extraction was performed by at least two reviewers (AC, ER, KA), with disputes resolved by discussion with the third. Data were recorded regarding study characteristics, patient demographics, stages studied, ctDNA collection method, the timing of ctDNA collection, and reported recurrence-free survival (RFS)/recurrence-free interval (RFI) in both post-surgical and post-adjuvant chemotherapy periods. In addition, data were recorded for individual subgroups, such as stages, and the study at large when available. 

### 2.3. Statistical Analysis

Data analysis was performed using Review Manager V.5.3 (The Nordic Cochrane Center, Cochrane Collaboration, Copenhagen, Denmark). If the study had more than one outcome, then the precision was compared to give a more conservative estimate of the HRs and 95% CI. The I^2^ statistic was used to assess the statistical heterogeneity. An I^2^ statistic of >50% was considered significant heterogeneity. Statistical significance was set at *p*-value < 0.05. Publication bias was assessed visually using funnel plots. All studies were assessed to be of moderate quality. The pooled HR and 95% CI are represented in forest plots. Each square on the chart area represents an individual study, and the area of each square is equivalent to the weight of the study, which is the inverse of the study variance. The diamond represents summary measures, and the width corresponds to the 95% CI. A random-effects model with inverse variance (DerSimonian and Laird method) was applied [22]. Heterogeneity was estimated using the inconsistency index and χ^2^ test.

## 3. Results

**Figure 1:**
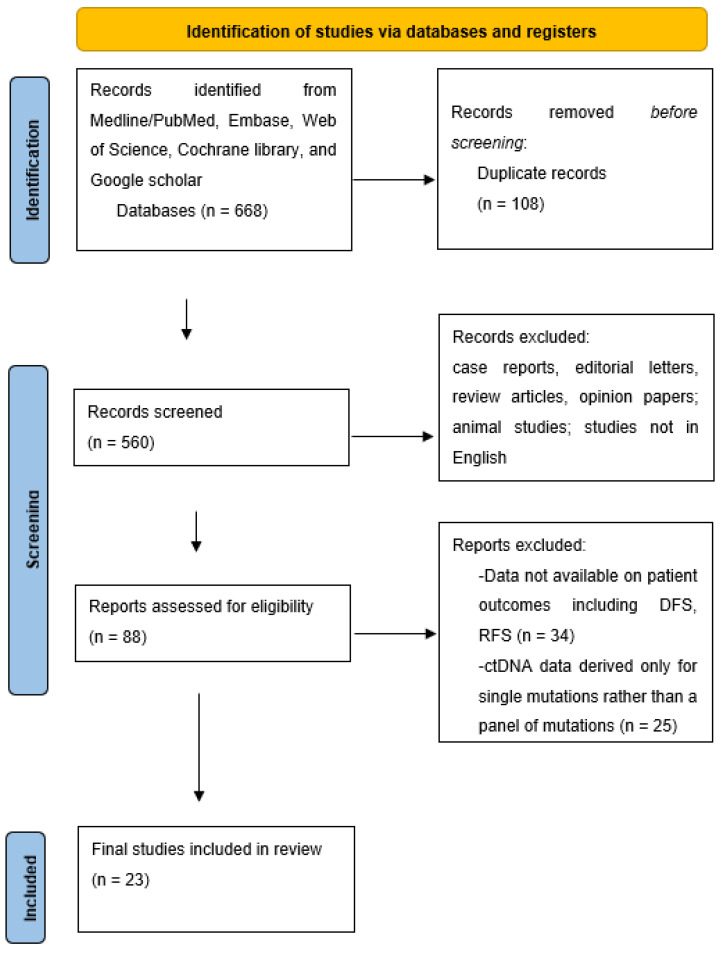
PRISMA flow diagram.

Our search yielded a total of 668 articles. After screening and final selection, 23 unique studies provided quantitative data on RFS based on the post-operative and post-adjuvant ctDNA status as shown in PRISMA diagram in Figure 1. The characteristics of these studies are summarized in Table 1. Henricksen et al., 2021, and Henricksen et al., 2022, were duplicates but were used for different analyses [20,21]. Of these studies, seven provided data on the prognostic value of post-adjuvant ctDNA. The studies primarily focused on locally invasive or otherwise nonmetastatic cancers, although eight studied ctDNA in oligometastatic stage IV rectal cancer amenable to curative-intent resection. Most studies (17/23) utilized a tumor-informed ctDNA analysis method. 

The data comprised 3568 patients. Of this population, 13.4% (477) were positive for ctDNA post-operatively. Likewise, 1007 patients were assessed in the post-adjuvant setting.

**Figure 2:**
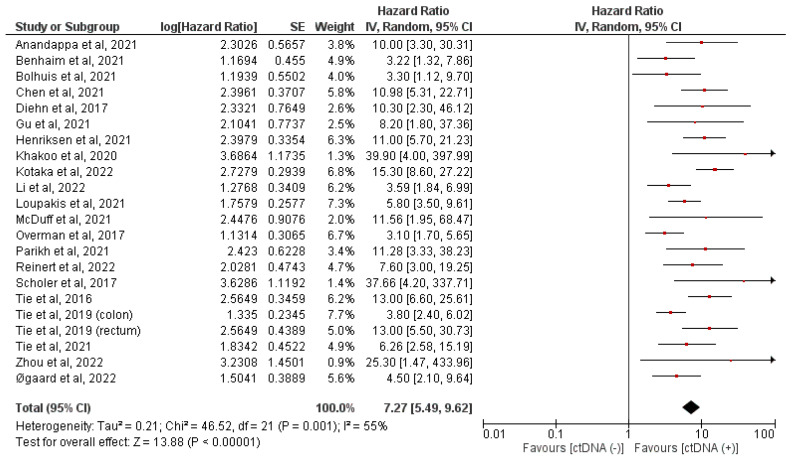
Forest plot showing the pooled hazard ratio based on post-surgical ctDNA-positive versus ctDNA-negative status. The hazard ratio for each adverse event is represented by a square, and the horizontal lines crossing the squares represent the 95% confidence interval (CI) [9,18,20,21,23,24,25,26,27,28,29,30,31,32,33,34,35,36,37,38,39,40,41].

**Figure 3:**
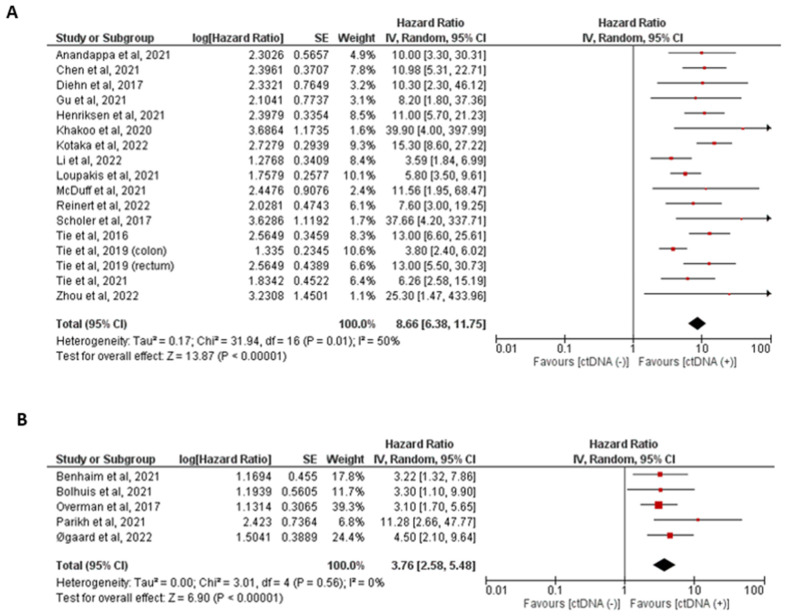
Forest plots showing the pooled hazard ratio based on ctDNA method: (**A**) post-surgical ctDNA positive versus ctDNA negative status via tumor-informed method; (**B**) post-surgical ctDNA positive versus ctDNA negative status via tumor-agnostic method. The hazard ratio for each adverse event is represented by a square, and the horizontal lines crossing the squares represent the 95% confidence interval (CI) [9,18,20,21,23,24,25,26,27,28,29,30,31,32,33,34,35,36,37,38,39,40,41].

Utilizing a random-effects model, analysis of our primary outcome, and post-surgical ctDNA status showed a statistically significant prognostic effect (pooled HR = 7.27 (95% CI 5.49–9.62, *p* < 0.0001)). This indicates that the presence of positive ctDNA results after surgery yields a poor prognosis. A forest plot of these data is shown in Figure 2. These data had moderate heterogeneity (I^2^ = 55%). Subgroup analyses were performed on these data, as shown in Table 2. There have been insufficient studies to stratify post-adjuvant ctDNA results for oligometastatic stage IV disease and tumor-agnostic methodologies. Among these analyses, all the pooled hazard ratios reached significance. Heterogeneity was improved when stratifying by the tumor-informed versus tumor-agnostic ctDNA collection method, especially in the tumor-informed group. Similarly, heterogeneity improved when only oligometastatic stage IV was analyzed. Forest plots of tumor-agnostic and tumor-informed ctDNA statuses are shown in Figure 3.

**Figure 4:**
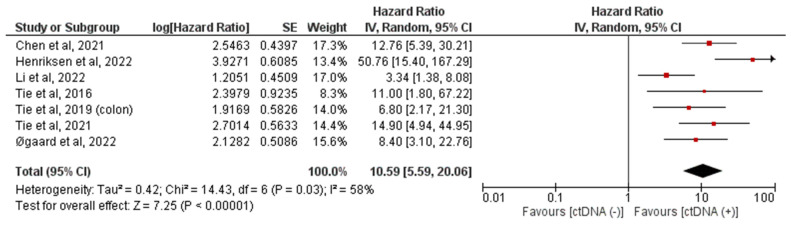
Forest plot showing the pooled hazard ratio based on the ctDNA method based on post-adjuvant ctDNA positive versus ctDNA-negative status. The hazard ratio for each adverse event is represented by a square, and the horizontal lines crossing the squares represent the 95% confidence interval (CI) [18,21,26,31,34,39,40].

Similarly, a random-effects model was used to calculate the pooled HR for the ctDNA status in the post-adjuvant chemotherapy setting, which also yielded a statistically significant result, that positive ctDNA implies a higher risk of recurrence (pooled HR = 10.59 (95% CI 5.59–20.06)). The pooled hazard ratio based on the ctDNA method based on post-adjuvant ctDNA-positive versus ctDNA-negative status is shown in Figure 4. Unfortunately, a meta-analysis could only be performed on stages I–III and with the tumor-informed methodology in the post-adjuvant setting owing to the smaller number of studies. 

## 4. Discussion

Our study demonstrated that patients with ctDNA-positive status after curative-intent surgery were significantly associated with low RFS (pooled HR = 7.27 (95% CI 5.49–9.62, *p* < 0.0001)). This indicates that patients with positive ctDNA following curative-intent surgery have a poorer prognosis than ctDNA-negative patients. Based on these results, ctDNA analysis can reliably identify patients at a higher risk of recurrence and those who can benefit from adjuvant systemic treatments. This could spare patients from unnecessary or inappropriate toxic treatments. Therefore, ctDNA analysis could also be used as a predictive marker. A phase II/III study, NRG-GI005 (COBRA), is currently testing whether ctDNA can be a predictive biomarker for adjuvant chemotherapy benefit in patients with resected stage II colon cancer [42].

After practicing for decades with no reliable minimally invasive marker, the practice is changing to now include post-operative ctDNA analysis to help guide our decisions regarding adjuvant therapy. Our study is the largest meta-analysis to explore the role of ctDNA assay. A smaller meta-analysis of seven studies with data prior to 2019 included 424 patients and showed a statistically significant association between post-surgical ctDNA and RFS [43]. The current prospective studies with ctDNA have a small number of patients and do not reflect the true value of ctDNA in MRD monitoring [40]. Our meta-analyses included 23 studies with 3568 patients [9,18,20,21,23,24,25,26,27,28,29,30,31,32,33,34,35,36,37,38,39,40,41,44,45]. This speaks to the rapidly expanding number of studies on the topic. Synthesizing an emerging abundance of robust data is essential. 

We performed subgroup analyses of patients based on the colorectal cancer stage. Patients with stage I–III CRC are eight times more likely to recur with positive ctDNA results than ctDNA-negative patients. This provides an indicator for patients who may benefit from further adjuvant treatment to prevent recurrence. Further studies on this topic are ongoing. CIRCULATE-Japan, which encompasses three clinical trials, is currently examining the clinical benefits of ctDNA analysis and adjuvant treatment in patients with resectable colorectal cancer [30,46].

Our analysis also showed that patients who had positive ctDNA after receiving adjuvant chemotherapy had a poorer prognosis with lower RFS than ctDNA-negative comparators (HR 10.59, 95% CI 5.59–20.06). Post-adjuvant ctDNA levels could be used to determine the risk of recurrence and the need for further close surveillance [47,48]. For example, nearly half of the patients with stage IV CRC with liver oligometastases recur after curative-intent surgery. Reinert and colleagues studied these patients with serial ctDNA studies in addition to routine surveillance imaging [36]. The study showed that ctDNA detected recurrence with a median time of 2.5 months (*p* < 0.0001) prior to routine surveillance imaging, especially in those with indeterminate CT findings. This indicates that ctDNA can be used as a surveillance tool to assess recurrence. In a study of 138 patients with metastatic gastrointestinal cancer, Parikh et al. found that serial ctDNA monitoring could predict the response to systemic treatment [49,50]. Currently, the NRG-GI008 trial is recruiting patients with stage III and high-risk stage II colon cancer to determine which patients benefit from adjuvant chemotherapy based on the circulating tumor DNA results [51].

We also explored the role of different ctDNA analysis methods on prognostications, as previous studies have shown that tumor-informed methods are more sensitive and specific compared to tumor-agnostic methods. We performed a subgroup analysis of ctDNA analysis methods in the post-surgical setting. This demonstrated that ctDNA positivity using tumor-informed and tumor-agnostic methods was associated with low RFS, with pooled hazard ratios of 8.66 (95% CI 6.38–11.75) and HR = 3.76 (95% CI 2.58–5.48), respectively. While these data indicate better prognostication for tumor-informed methodologies in line with previously published information, the studies included did not include head-to-head analysis, and so conclusive arguments are difficult to make from these data. However, this is consistent with previously published results, which showed that studies that used tumor-informed assays showed higher rates of recurrences than those that used tumor-agnostic assays.

Monitoring ctDNA levels in the blood has been shown to accurately detect MRD and aid in measuring the therapeutic effects after curative treatment. While ctDNA is not yet the standard of care in clinical practice for CRC patients, studies are ongoing to define the appropriate way to use it as a tool in the clinic [19,52,53]. In 2022, a phase two randomized trial, Circulating Tumor DNA Analysis Informing Adjuvant Chemotherapy (DYNAMIC), showed non-inferiority in the 2-year recurrence-free survival between the standard management group and ctDNA-guided management in stage II CRC patients after curative-intent surgery (93.5% vs. 92.4%, 95% CI [−4.1 to 6.2], non-inferiority margin, −8.5 percentage points) [48]. Building upon the findings of this study, which suggested the sparing of adjuvant therapy in post-surgery ctDNA-negative stage II colon cancer patients, our study further supports the notion that tumor-informed ctDNA analysis may offer enhanced reliability compared to the tumor-agnostic approach. To the best of our knowledge, this is the largest meta-analysis to confirm the prognostic and predictive power of ctDNA levels in the post-operative and post-adjuvant chemotherapy settings. 

ctDNA studies on surgically treated colorectal carcinomas have consistently demonstrated excellent reproducibility, underscoring the reliability and robustness of this approach. Numerous studies have provided compelling evidence of the reproducibility of ctDNA analysis in detecting minimal residual disease and monitoring disease recurrence. These findings highlight the potential of ctDNA as a valuable tool for post-operative surveillance in colorectal cancer patients. Furthermore, it is noteworthy that the prognostic significance of ctDNA studies has been consistently reported across various investigations, further reinforcing its clinical relevance. The high reproducibility and prognostic value of ctDNA analysis in surgically treated colorectal carcinomas support its potential as a non-invasive, reliable biomarker for post-operative monitoring and risk stratification.

Patients with peritoneal carcinomatosis or brain metastases, for instance, pose difficulties due to the plasma–peritoneal and blood–brain barriers impending accurate ctDNA detection. These limitations necessitate careful consideration when applying liquid biopsy techniques in such contexts. Additionally, to overcome the challenges associated with ctDNA analysis and maximize its clinical utility, it is imperative to establish standardized measures for ctDNA profiling across different platforms. The lack of uniformity in the current methodologies and technologies necessitates a concerted effort to achieve consistency and comparability in ctDNA assessment. By implementing robust protocols and harmonizing analytical approaches, the reliability and accuracy of ctDNA profiling can be significantly improved, facilitating its integration into routine clinical practice.

### Limitations

Abstracts with insufficient or imprecise data were excluded. Studies that included stage 0 CRC were omitted if they lacked subgroup analysis excluding this population. The sensitivity and specificity of the ctDNA methods are different. We attempted to overcome this by performing subgroup analyses of tumor-naive vs. tumor-informed techniques. Multiple abstracts were published on the same population at different time points during follow-up, so we eliminated the duplicates by reviewing all abstracts and manuscripts in detail and included only the most recent abstracts or manuscripts with the greatest patient populations. The numbers of mutational gene panels tested were different with different methods, and so the mean depth of the sequencing yield is different as well. Because this is a new technique, the follow-up was not very long for some studies. There are also limited studies for certain populations that prevent meta-analyses from being performed. Additionally, oligometastatic colorectal cancer patients were found to have a lower hazard ratio than earlier-stage cancers, which could be secondary to the limited number of studies in this setting. 

## 5. Conclusions

Our study is the largest and most up-to-date meta-analysis of studying the effect of ctDNA status in both post-curative-intent surgery and post-adjuvant chemotherapy in CRC. Our study validated the role of ctDNA analysis in stage I–oligometastatic stage IV colorectal cancer patients. Our analysis emphasizes that post-operative ctDNA is a strong prognostic marker of RFS. Based on our results, ctDNA can be a significant and independent predictor of RFS. This real-time assessment of treatment benefits can be used as a surrogate endpoint for the development of novel drugs. Few ctDNA-based clinical trials are ongoing internationally to confirm the clinical utility of ctDNA in colorectal cancer. Further randomized clinical trials, in which ctDNA results are used to inform patient management, are required to assess the clinical utility of ctDNA-guided approaches for colorectal cancer management and surveillance.

### Future Directions

The potential of ctDNA analysis to guide treatment decisions in cancer patients, particularly in the context of adjuvant therapy, holds great promise. Looking ahead, it is essential that future prospective clinical trials on colorectal cancer and other gastrointestinal malignancies incorporate baseline ctDNA collection to gain a better understanding of the tumor shed rate, tumor fraction, MRD, and molecular heterogeneity. We propose that future clinical studies should prioritize the following criteria when considering the omission of adjuvant therapy: (1) a comprehensive evaluation of the ctDNA status using tumor-informed methods; (2) rigorous stratification of patients based on their disease stage; and (3) meticulous assessment of the prognostic reliability of post-surgical ctDNA analysis in patients of different stages. By adhering to these criteria, future studies can contribute to the establishment of evidence-based guidelines for treatment decision making, facilitating personalized approaches and potentially sparing patients unnecessary adjuvant therapy while maintaining optimal outcomes. Longitudinal testing is crucial for monitoring recurrence, assessing the treatment response, and detecting resistance alterations, which can increase the sensitivity of ctDNA testing. However, the timing of MRD testing should be considered, and it is recommended to perform testing four weeks post-operatively to avoid interference from cell-free DNA. In addition, there is a need to establish consensus minimum standards for ctDNA specimen collection and processing in clinical trial protocols to achieve harmonization across studies and facilitate cross-study analyses. By implementing these standards, the reliability and reproducibility of ctDNA-based clinical trials can be improved, and the development of personalized cancer therapies can be accelerated. Practical considerations for ctDNA collection and processing must be a priority for future studies in colorectal cancer.

## Figures and Tables

**Table 1:** Characteristics of the studies included in this meta-analysis.

Study	Stage of CRC	Colon vs. Rectal Cancer	Type of ctDNA Assay	Tumor-Informed vs. Tumor-Agnostic/Naive	Timing of ctDNA Collection	Number of Patients	Number of ctDNA-Positive Patients
Anandappa et al., 2021 [23]	II–III	CRC	mPCR	Informed	NA	107	14 (13%)
Benhaim et al., 2021 [24]	II–III	CRC	QiAamp	Naive	5 days	187	18 (9%)
Bolhuis et al., 2021 [25]	Stage IV OM	Colon	dd-PCR	Naive	~38 days		6 (26%)
Chen et al., 2021 [26]	II–III	CRC	Geneseeq Prime	Informed	3–7 days	240	20 (8%)
Diehn et al., 2017 [27]	II–III	CRC	AVENIO	Informed	10 days	145	12 (8%)
Gu et al., 2021 [28]	I–III	CRC	Super-Seq	Informed	7–10 days	25	4 (16%)
Henriksen et al., 2021 [20] (stages I–III)	I–III	CRC	Signatera, bespoke mPCR NGS assay	Informed	2–4 weeks	218	20 (9%)
Henriksen et al., 2022 [21]	III	CRC	Signatera, bespoke mPCR NGS assay	Informed	2–4 weeks	NA (numbers already included in the above study)	NA
Khakoo et al., 2020 [29]	I–III	Rectal	dd-PCR	Informed	4–12 weeks	47	3 (6%)
Kotaka et al., 2022 [30]	I–Stage IV OM	CRC	Signatera, bespoke	Informed	4 weeks	1365	115 (8%)
Li et al., 2022 [31]	III	Colon	AVENIO	Informed	2–4 weeks	151	24 (15%)
Loupakis et al., 2021 [32]	Stage IV OM	CRC	Bespoke mPCR	Informed	8–99 days	112	61 (54%)
McDuff et al., 2021 [33]	II–III	CRC	dd-PCR	Informed	1–5 months	19	4 (21%)
Øgaard et al., 2022 [34]	Stage IV OM	CRC	TriMeth	Naive	0.9–1.7 months	96	39 (40%)
Overman et al., 2017 [35]	Stage IV OM	CRC	Guardant Reveal	Naive	Immediately post-op	54	24 (44%)
Parikh et al., 2021 [9]	I–Stage IV OM	CRC	Guardant Reveal NGS	Naive	4 weeks	84	17 (20%)
Reinert et al., 2022 [36]	Stage IV OM	CRC	dd-PCR	Informed	30 days	40	13 (32%)
Tie et al., 2017 [37]	I–Stage IV OM	CRC	dd-PCR	Informed	0, 8, 30 days, 3 months	27	6 (22%)
Tie et al., 2016 (stage II) [18]	II	Colon	Safe-SeqS	Informed	4–10 weeks	230	20 (8%)
Tie et al., 2019 (colon) [38]	III	Colon	Safe-SeqS	Informed	4–10 weeks	96	20 (20%)
Tie et al., 2019 (rectum) [39]	II–III	Rectal	Safe-SeqS	Informed	4–10 weeks	159	19 (11%)
Tie et al., 2021 [40]	Stage IV OM	CRC	Safe-SeqS	Informed	4–10 weeks	54	12 (22%)
Zhou et al., 2022 [41]	II–III	Rectal	QiAMP	Informed	<1 month	89	6 (6%)

CRC: colorectal cancer; OM: oligometastatic; NA: not available; mPCR: multiplex PCR; dd-PCR: droplet digital PCR; RCT: randomized control trial.

**Table 2:** Pooled hazard ratio for subgroup analyses based on stage of CRC and method of ctDNA analysis.

Subgroup	Pooled HR (CI)	Number of Studies
Post-surgical	7.27 (95% CI 5.49–9.62)	22
Stage		
I–III	8.14 (95% CI 5.60–11.82)	14
IV oligometastatic	4.83 (95% CI 3.64–6.39)	8
ctDNA Method		
Tumor-Informed	8.66 (95% CI 6.38–11.75)	17
Tumor-Agnostic	3.76 (95% CI 2.58–5.48)	5
Post-Adjuvant	10.59 (95% CI 5.59–20.06)	7
Stage		
I–III	10.60 (95% CI 4.21–26.69)	5
IV oligometastatic	NA	2
ctDNA Method		
Tumor-Informed	11.16 (95% CI 5.19–23.98)	6
Tumor-Agnostic	NA	1

## Data Availability

Not applicable.
